# Frequency-dependent transition in power-law rheological behavior of living cells

**DOI:** 10.1126/sciadv.abn6093

**Published:** 2022-05-06

**Authors:** Jiu-Tao Hang, Guang-Kui Xu, Huajian Gao

**Affiliations:** 1 Laboratory for Multiscale Mechanics and Medical Science, Department of Engineering Mechanics, SVL, School of Aerospace Engineering, Xi’an Jiaotong University, Xi’an 710049, China.; 2 School of Mechanical and Aerospace Engineering, College of Engineering, Nanyang Technological University, Singapore 639798, Singapore.; 3 Institute of High Performance Computing, A*STAR, Singapore 138632, Singapore.

## Abstract

Living cells are active viscoelastic materials exhibiting diverse mechanical behaviors at different time scales. However, dynamical rheological characteristics of cells in frequency range spanning many orders of magnitude, especially in high frequencies, remain poorly understood. Here, we show that a self-similar hierarchical model can capture cell’s power-law rheological characteristics in different frequency scales. In low-frequency scales, the storage and loss moduli exhibit a weak power-law dependence on frequency with same exponent. In high-frequency scales, the storage modulus becomes a constant, while the loss modulus shows a power-law dependence on frequency with an exponent of 1.0. The transition between low- and high-frequency scales is defined by a transition frequency based on cell’s mechanical parameters. The cytoskeletal differences of different cell types or states can be characterized by changes in mechanical parameters in the model. This study provides valuable insights into potentially using mechanics-based markers for cell classification and cancer diagnosis.

## Teaser

A self-similar hierarchical model can naturally reproduce power-law rheology of cells in both low- and high-frequency scales.

## INTRODUCTION

Studies of dynamical mechanical properties of cells are crucial for understanding cellular migration, differentiation, and carcinogenesis (*1*–*3*). Living cells are complex active materials with both solid-like elastic and fluid-like viscous properties. Many experiments (*4*–*10*) have shown that the complex modulus of cells is dependent on frequency. A notable feature is that regardless of cell types, cell states (e.g., drug-induced), or measuring methods, the complex moduli G^*^ exhibit a weak power-law dependence on frequency *f* in a relatively low-frequency range of 10^−2^ to 10^2^ Hz; i.e., G^*^ ~ (*if*)^α^ where $i=\sqrt{-1}$ (*4*–*10*). Meanwhile, the power-law exponent α of the storage modulus *G*′ is approximately the same as that of the loss modulus *G*″, both in the range of 0.1 to 0.3 in most experiments (*6*–*9*). However, as the frequency further increases, the weak power-law relationship breaks down, and the loss modulus *G*″ shows a greater dependence on frequency than the storage modulus *G*′ (*9*–*13*). For example, at high frequencies, the storage moduli of 3T3 fibroblast and neural cells do not vary with frequency (*10*, *12*), while the loss moduli still show a power-law dependence on frequency with an exponent ~1.0 (*9*, *10*, *12*). Recently, Rigato *et al*. (*10*) experimentally studied cell rheology at high frequencies (up to 10^5^ Hz) and found that benign (MCF10A) and malignant (MCF7) cancer cells exhibit distinctly different rheological properties at high frequencies. Currently, there is very little theoretical understanding of these rheological characteristics of cells over different frequency scales.

A number of models have been proposed to explain the power-law rheological behavior of cells. Nicolas *et al*. (*14*) and Bausch *et al*. (*15*) used a linear viscoelastic model consisting of two springs and two dashpots to depict the creep response of cells. The disadvantage of such a linear viscoelastic model is that it requires a large number of springs and dashpots to accommodate the power-law property without a clear physical interpretation (*16*, *17*). By adding a Newtonian viscous term into the soft glass rheology (SGR) theory, Fabry *et al*. (*9*) succeeded in explaining the weak power-law rheology of cells at low frequencies and realizing part of rheological characteristics at high frequencies, but the loss modulus based on SGR theory tends to 0 at high frequencies (*18*). A poroelastic material theory for modeling the relaxation behavior of cytoplasm (*19*) failed to accurately predict the power-law rheological characteristics of cells (*19*, *20*). These models provide a good description of the rheological behavior of cells in a limited frequency range (e.g., low frequencies), but they usually do not provide a mechanistic understanding and cannot capture the full phenomenology over the entire frequency range investigated (*17*). Some bottom-up models, such as those based on polymeric molecules, suggest multiple mechanisms to account for the stress-stiffening response of cytoskeletal networks, but they are less successful in modeling the weak power-law dependency of elastic moduli on frequency (*17*). So far, most studies on the rheological behavior of living cells have been focused on low frequencies, and relatively little is known about their rheological properties at high frequencies. In particular, the transition of power-law behaviors in living cells, which has the potential to distinguish between different cell states (e.g., cell carcinogenesis), is rarely investigated.

Here, we propose a self-similar hierarchical model to describe cell’s rheological behavior over a frequency range spanning several orders of magnitudes. At low frequencies, our model naturally reproduces the weak power-law dependence on frequency for both storage and loss moduli. At high frequencies, this model predicts that the complex modulus of cells no longer exhibits a simple power-law dependence on frequency, but instead the storage modulus tends to a constant, while the loss modulus becomes linearly proportional to the frequency. A transition frequency has been identified to distinguish between cell’s rheological differences under different frequency scales for different cell states or types. Using this model, we show that the transition frequency of malignant cancer cells is nearly five times higher than that of benign cancer cells. In addition, we compare the predictions of our model with other models, with results showing that the present model is in broad agreement with all existing experimental findings.

## RESULTS

### The self-similar hierarchical model of cells

**Fig. 1.**
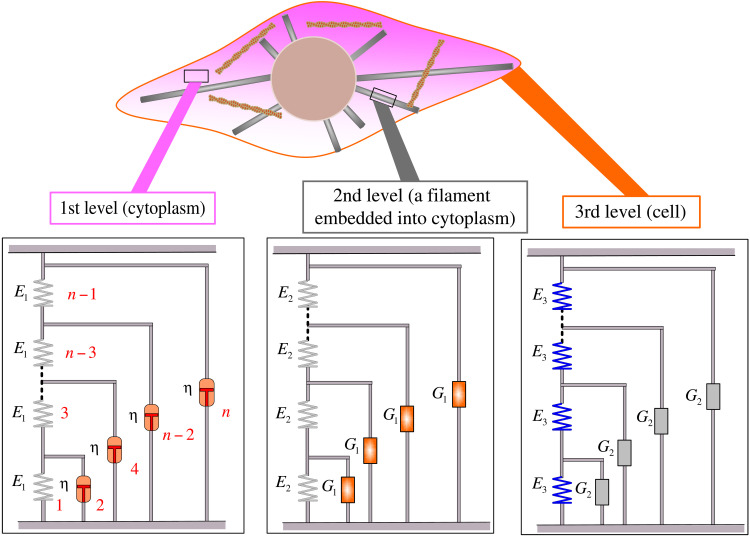
The self-similar hierarchical model of cells. The 1st-level hierarchy is constructed as a ladder-like structure where springs with stiffness *E*_1_ run along the struts and dashpots with viscosity η on the rungs of the ladder (elements are numbered in red). The 2nd-level hierarchy is constructed by having the 1st-level hierarchy as building block *G*_1_ along with springs of stiffness *E*_2_. The 3rd-level hierarchy is constructed by arranging the 2nd-level hierarchy as building block *G*_2_ along with springs of stiffness *E*_3_. In this model, the cytoplasm serves as the 1st-level hierarchy that fills the entire cell; the cytoskeletal filament (e.g., microtubule) embedded into the cytoplasm is considered the 2nd-level hierarchy; and lastly, the entire cell is modeled as the 3rd-level hierarchy.

Many power-law phenomena in nature and human behavior are inseparable from the self-similarity principle (*21*–*23*). On the basis of the multilevel structural features of cells, we propose a self-similar hierarchical model to describe their rheological behavior. Cells are complex structures consisting of cytoplasm, cytoskeleton, and membrane. Because the cytoplasm is ubiquitous with both solid and fluid properties, we discretize its spatial solid component into an infinite number of springs with elastic stiffness *E*_1_ in series immersed in a viscous fluid with a viscous coefficient η (see Fig. 1), which forms the 1st-level hierarchy (*G*_1_). Cytoskeletal filaments are composed of monomers that are strung together end to end and are embedded into the cytoplasm. A single cytoskeletal filament is thus discretized into a series of tandem springs with elastic stiffness *E*_2_ embedded in the cytoplasm, which serves as the 2nd-level hierarchy (*G*_2_), with the 1st-level hierarchy as a building block (see Fig. 1). The whole-cell structure, as an intricate three-dimensional network consisting of emanative microtubules, actin filaments, and so on (*3*), is treated as a series of individual filaments connected by springs (the transverse expansion of the cytoskeleton) with elastic stiffness *E*_3_, which forms the 3rd-level hierarchy (*G*_3_), with the 2nd-level hierarchy as a building block (see Fig. 1). In this way, the previous level hierarchy is embedded into the current level as a building block, making each level similar in form and the whole system self-similar. At the whole-cell level (the 3rd-level hierarchy), there are a total of four mechanical parameters: *E*_1_ represents the effective stiffness of the cytoplasm, η represents the effective viscosity of the cytoplasm, *E*_2_ represents the effective stiffness of the cytoskeletal filaments, and *E*_3_ represents the effective stiffness of the transverse expansion of the cytoskeletal network.

### The complex modulus of the proposed model

Let $G_{1}^{*}$, $G_{2}^{*}$, and $G_{3}^{*}$ represent the complex moduli of the 1st-, 2nd-, and 3rd-level hierarchies, respectively. When the number *n* of elements in each hierarchy approaches infinity, increasing elements will not change the complex modulus, that is, $G_{i}^{*}(n-2)=G_{i}^{*}(n)$ (*i* = 1, 2, and 3). Thus, the complex modulus of the 1st-level hierarchy can be obtained by solving the equation ${\left(G_{1}^{*}\right)}^{2}-i\omega \eta G_{1}^{*}-i\omega \eta E_{1}=0$, where ω is the angular frequency (ω = 2π*f*). This leads to the following solution

$$G_{1}^{*}=\frac{i\omega \eta +\sqrt{{\left(i\omega \eta \right)}^{2}+4i\omega \eta E_{1}}}{2}$$ (1)

Replacing η by the characteristic relaxation time τ = η/*E*_1_ yields

$$G_{1}^{*}=E_{1}\frac{1+\sqrt{1+4{\left(i\omega \tau \right)}^{-1}}}{2{\left(i\omega \tau \right)}^{-1}}$$ (2)

Similarly, the complex moduli of 2nd- and 3rd-level hierarchies are obtained as

$$G_{2}^{*}=\frac{G_{1}^{*}+\sqrt{{\left(G_{1}^{*}\right)}^{2}+4E_{2}G_{1}^{*}}}{2}$$ (3)

$$G_{3}^{*}=\frac{G_{2}^{*}+\sqrt{{\left(G_{2}^{*}\right)}^{2}+4E_{3}G_{2}^{*}}}{2}$$ (4)

### Dynamical mechanical behavior of cells at both low and high frequencies

Experiments have shown that the rheological characteristics of cells vary notably from low to high frequencies (*9*–*12*). Therefore, understanding the cell’s rheological properties in different frequency ranges is of great significance for cell classification, identification, and diagnosis. Here, by using the proposed model, we analyze the rheological characteristic of cells spanning several orders of magnitudes of frequency. At low frequencies (i.e., ωτ < < 1), the complex moduli of the self-similar hierarchical model can be approximated as

$$\underset{\omega \tau \to 0}{\text{lim}}G_{1}^{*}=E_{1}(i\omega \tau )/2+E_{1}{\left(i\omega \tau \right)}^{0.5}$$ (5)

$$\underset{\omega \tau \to 0}{\text{lim}}G_{2}^{*}=G_{1}^{*}/2+{\left(E_{2}G_{1}^{*}\right)}^{0.5}$$ (6)

$$\underset{\omega \tau \to 0}{\text{lim}}G_{3}^{*}=G_{2}^{*}/2+{\left(E_{3}G_{2}^{*}\right)}^{0.5}$$ (7)

Because ωτ < < 1, the power-law exponent of the 1st-level hierarchy approaches 0.5, and the lower bounds of power-law exponents of the 2nd- and 3rd-level hierarchies are 0.25 and 0.125, respectively. Therefore, at low frequencies, the complex modulus of the entire cell (the 3rd-level hierarchy) exhibits a weak power-law dependence on the frequency with the power-law exponents of its storage and loss moduli being approximately equal, as in our previous work (*24*). However, at high frequencies (i.e., ωτ > > 1), the complex moduli of the self-similar hierarchical model become

$$\underset{\omega \tau \to \infty }{\text{lim}}G_{1}^{*}=\frac{E_{1}(i\omega \tau )}{2}[2+\sqrt{1+\frac{4}{i\omega \tau }}-1]=i\omega \tau E_{1}+E_{1}$$ (8)

$$\underset{\omega \tau \to \infty }{\text{lim}}G_{2}^{*}=i\omega \tau E_{1}+E_{1}+E_{2}$$ (9)

$$\underset{\omega \tau \to \infty }{\text{lim}}G_{3}^{*}=i\omega \tau E_{1}+E_{1}+E_{2}+E_{3}$$ (10)

It is clear that when ωτ is sufficiently high, the power-law exponents of loss moduli $G_{i}^{\prime \prime }$ at each level of the hierarchy are all close to 1.0, while the storage moduli $G_{i}^{\prime }$ at each level of the hierarchy are all constants. These predictions are in excellent agreement with recent experiments (*9*, *10*, *12*). Note that, in the limit of infinite frequency, the storage modulus becomes a constant equal to the sum of elastic moduli from each level of the hierarchy, and all of these are predicted on the assumption that the spring stiffnesses remain constant under loading.

**Fig. 2.**
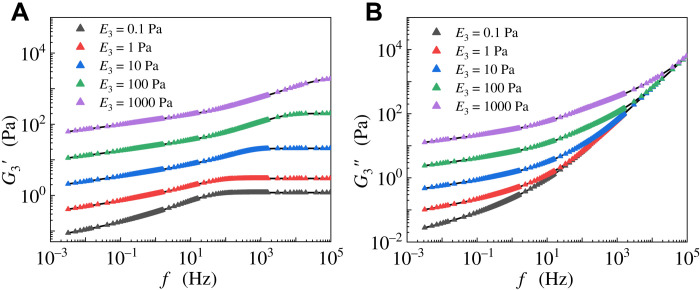
Comparison between analytical (Eq. 4) and numerical solutions. The solid lines and scatter points represent analytical and numerical results, respectively. The (**A**) storage and (**B**) loss moduli of the 3rd-level hierarchy versus angular frequency for different values of *E*_3_.

Figure 2 shows the analytical (Eq. 4) and numerical solutions for different values of *E*_3_. It can be seen that both storage and loss moduli exhibit a weak power-law dependence on frequency in the low-frequency range, and the storage modulus tends to a constant, while the loss modulus becomes linearly proportional to frequency in the high-frequency range. These results are consistent with Eqs. 7 and 10. On the basis of the above analysis, the power-law exponents of the 1st-, 2nd-, and 3rd-level hierarchies are in the ranges of 0.5 to 1.0, 0.25 to 1.0, and 0.125 to 1.0 under different frequency scales, respectively. Notably, the 3rd-level of the self-similar hierarchical model can cover all power-law exponents of cell rheology reported in different frequency scales (*4*, *6*–*10*, *17*).

### Rheological behavior of different cells in different frequency scales

**Fig. 3.**
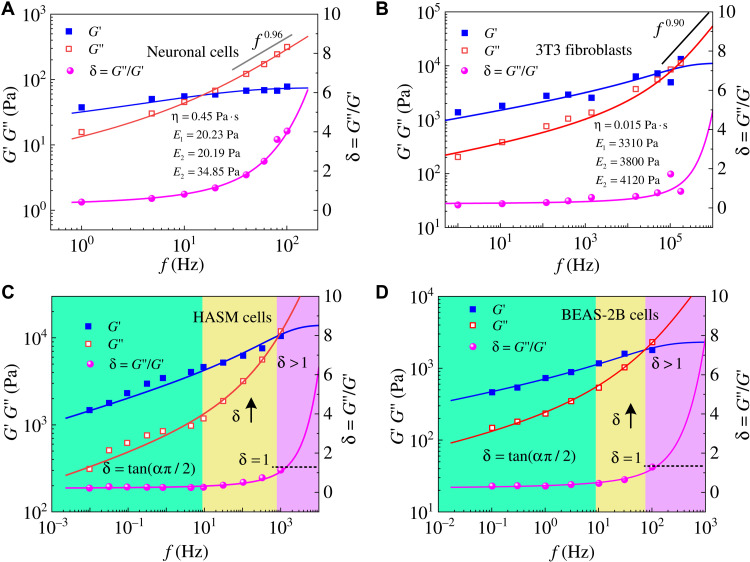
Cells exhibit different rheological behavior at different frequency scales. Our proposed self-similar hierarchical model can realize the rheological characteristics of different types of cells in different frequency ranges. Both storage and loss moduli of (**A**) neuronal cells (*12*) and (**B**) 3T3 fibroblasts (*10*) are consistent with our models. The predictions of our model (solid line) fit well with experimental data of (**C**) HASM cells (*9*) and (**D**) bronchial (BEAS-2B) epithelial cells (*25*) in a vast frequency range. Depending on the loss tangent δ, the complex moduli of cells can be divided into three regions, indicated by green (region I), yellow (region II), and purple (region III), respectively.

Gong *et al*. (*12*) and Rigato *et al*. (*10*) used atomic force microscopy to obtain the complex moduli of neural (Fig. 3A) and 3T3 fibroblast (Fig. 3B) cells, respectively, both of which showed a steeper rise in loss modulus at high frequencies. Different from existing models, our model can fit these experimental data very well over the entire frequency scales. The power-law exponent of loss modulus *G*″ of neural cells approaches 1.0 between 10^1^ and 10^2^ Hz (*12*), while 3T3 fibroblast cells exhibit this rheological characteristic at a frequency higher than 10^5^ Hz (*10*). This is not unexpected, as neuronal cells are extremely soft, and thus their “high frequency” is lower than that of the 3T3 fibroblasts.

The ratio of loss modulus to storage modulus δ = *G*″/*G*′ is defined as the loss tangent. In lower-frequency ranges, the storage and loss moduli exhibit a weak power-law dependence on the frequency with similar power-law exponents, as reported in our model and many experiments (*4*, *6*–*10*, *17*). We can thus define δ at low frequencies as

$$\delta_{L}=\frac{\text{sin}(\alpha_{2}\pi /2)}{\text{cos}(\alpha_{1}\pi /2)}$$ (11)

Here, α_1_ and α_2_ represent the power-law exponents of storage and loss moduli at low frequencies, respectively. If α_1_ = α_2_, then one has δ_L_ = tan (απ/2) with α = α_1_ = α_2_. It should be noted that, although the power-law exponents of storage and loss moduli can have some differences, they are often regarded as having approximately the same value at low frequencies (*16*, *17*). However, when the frequency is sufficiently high, the storage modulus tends to a constant, while the loss modulus becomes linearly proportional to the frequency. According to Eq. 10, the loss tangent in the high-frequency range behaves as

$$\delta_{H}=\frac{\omega \eta }{E_{1}+E_{2}+E_{3}}$$ (12)

which increases linearly with frequency. As illustrated in Fig. 3 (C and D), we analyze the experimental data of human airway smooth muscle (HASM) cells in the frequency range of 10^−2^ to 10^3^ Hz (*9*) and bronchial (BEAS-2B) epithelial cells in the frequency range of 10^0^ to 10^5^ Hz (*25*) by using the frequency-dependent loss tangent δ values. It can be seen that the rheological behavior of cells in the entire frequency range can be divided into three regions: a constant value of loss tangent δ (region I), a loss tangent δ greater than 1 (region III), and a transition region (region II). When the frequency is relatively low (10^−2^ to 10^1^ Hz for HASM cells and 10^−1^ to 10^1^ Hz for BEAS-2B cells), the complex modulus G^*^ follows a weak power-law dependence on frequency [G^*^ ~ (*if*)^α^] with exponents of 0.16 for HASM cells and 0.20 for BEAS-2B cells. As reported, the power-law exponents of cells at low frequencies are between 0.1 and 0.3 (*9*–*11*, *25*). In this region, the loss tangent δ is a constant. As the frequency increases further (10^1^ to 10^3^ Hz for HASM cells and 10^1^ to 10^2^ Hz for BEAS-2B cells), the loss tangent δ begins to rise, meaning that the loss modulus increases faster than the storage modulus. When the frequency is sufficiently high (>10^3^ Hz for HASM cells and >10^2^ Hz for BEAS-2B cells), the loss modulus becomes higher than the storage modulus, i.e., δ > 1. Furthermore, in region III, the storage modulus becomes constant, while the loss modulus shows a power-law dependence on the frequency with an exponent equal to 1.0 (see Fig. 3, A and B).

### Dynamical mechanical changes of drug-treated cells

**Fig. 4.**
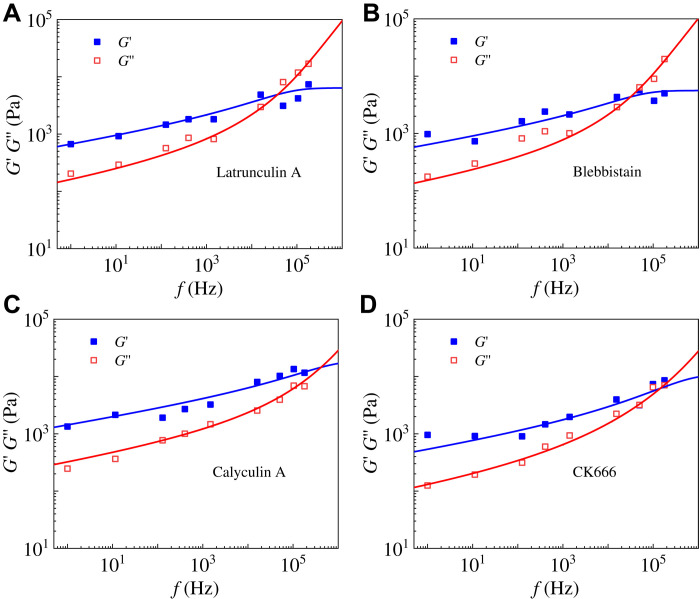
The predictions of our self-similar hierarchical model are in agreement with the experimental data (*10*) of 3T3 fibroblast cells treated with different drugs. Frequency-dependent storage and loss moduli of cells treated with the drug (**A**) latrunculin A, (**B**) blebbistatin, (**C**) calyculin A, and (**D**) CK666. The obtained mechanical parameters are in accord with drug-induced biological changes, as shown in Table 1.

Many experiments showed that the cytoskeleton plays a significant role in the dynamical mechanical properties of cells (*10*, *26*). Recently, Rigato *et al*. (*10*) experimentally studied the rheological behavior of 3T3 fibroblast cells in a vast frequency range by treating them with four different drugs. Figure 4 (A to D) shows that the predictions of our model are in excellent agreement with their experimental results. Table 1 lists the mechanical parameters, obtained by our model, of 3T3 fibroblasts under different drug treatments. The changes in mechanical properties can be understood as follows. As well known, the disruption of the cytoskeleton by latrunculin A and blebbistatin will decrease the stiffness of cells but increase their viscosity (*10*). As a result, the obtained elastic stiffnesses *E*_1_, *E*_2_, and *E*_3_ of cells treated with latrunculin A and blebbistatin are smaller than those of untreated ones, while the viscous coefficients η increase (see Table 1). The drug calyculin A can increase the intracellular tension (*27*), which would increase the cell stiffness and decrease its viscosity. Thus, the obtained elastic stiffnesses *E*_1_, *E*_2_, and *E*_3_ in this case should increase, while the value of η decreases significantly (see Table 1). Reduced actin cross-linking by the drug CK666 (*28*) will decrease the cytoskeleton stiffness, and the depolymerized actin components can increase the elastic stiffness of the cytoplasm (1st-level hierarchy). Meanwhile, the cytoplasmic viscosity will be reduced because of the architectural changes of the actin cortex from mesh-like to parallel arrangements (*28*). These biological changes coincide with our model predictions that the values of *E*_2_, *E*_3_, and η decrease and the value of *E*_1_ increases for CK666-treated cells (see Table 1).

**Table 1.** Mechanical parameters of different drug-treated 3T3 fibroblasts obtained by our model.

	η **(Pa·s)**	*E*_1_ **(kPa)**	*E*_2_ **(kPa)**	*E*_3_ **(kPa)**
Untreated	0.015 ± 10^−4^	3.31 ± 0.17	3.80 ± 0.37	4.12 ± 0.23
Latrunculin A	0.0265 ± 10^−4^	2.25 ± 0.12	2.36 ± 0.36	1.78 ± 0.06
Blebbistatin	0.0284 ± 10^−4^	1.76 ± 0.16	1.87 ± 0.44	1.97 ± 0.25
Calyculin A	0.0072 ± 10^−4^	6.44 ± 0.19	6.45 ± 0.53	5.99 ± 0.26
CK666	0.0087 ± 10^−4^	7.85 ± 0.07	1.51 ± 0.09	1.45 ± 0.04

Rigato *et al*. (*10*) assumed a double power-law model G*(*f*/*f*_0_) = *A*(*if*/*f*_0_)^α^ + *B*(*if*/*f*_0_)^β^ to describe the power-law rheological characteristics in low- and high-frequency ranges. They defined a transition frequency *f*_t_ when the weak power-law term is equal to the strong term [i.e., *A*(*f*_t_)^α^ = *B*(*f*_t_)^β^]. Staunton *et al*. (*29*) used a crossover frequency at which the loss tangent δ = 1 to indicate a transition from more solid-like (δ < 1) to more liquid-like (δ > 1) material properties. To better describe the rheological differences of cells in different frequency scales, we propose a transition frequency *f*_T_ when δ_L_ = δ_H_. In low-frequency ranges, δ_L_ = tan(απ/2) is a constant (*7*, *17*), and thus, the transition frequency is analytically expressed as

$$f_{T}=\frac{\left(E_{1}+E_{2}+E_{3})\text{tan}(\alpha \pi /2\right)}{2\pi \eta }$$ (13)

From the above equation, we find that the transition frequency *f*_T_ is proportional to the total stiffness *E*_sum_ = *E*_1_ + *E*_2_ + *E*_3_ of cells and inversely proportional to the cytoplasmic viscosity η. Notably, our defined transition frequency is related to elastic and viscous properties of cells, which can be used to investigate mechanical changes of cells in different states. For cells treated with latrunculin A and blebbistatin, the total stiffness decreases, while the viscosity increases, leading to reduced transition frequencies (see Eq. 13 and Table 2). For cells treated with calyculin A, the increase of cytoskeletal stiffness and the decrease of cytoplasmic viscosity can lead to a threefold increase in the transition frequency in contrast to untreated cells (see Table 2). For cells treated with CK666, the sum of *E*_1_, *E*_2_, and *E*_3_ is approximately equal to that of untreated cells (see Table 1), while the corresponding power-law exponent is half of the untreated cells, resulting in the transition frequency approximately twice as large as that of untreated cells. Furthermore, the sum *E*_sum_ = *E*_1_ + *E*_2_ + *E*_3_, as the total elastic moduli of the cell, can be directly measured in experiments. The total elastic moduli obtained from indentation experiments are 5.0 to 11.2 kPa for 3T3 fibroblast cells (*30*, *31*) and 0.2 to 2 kPa for bronchial epithelial cells (*32*), which are in good agreement with our results (11.23 kPa for 3T3 fibroblast cells and 1.72 kPa for bronchial epithelial cells).

**Table 2.** The transition and crossover frequencies of 3T3 fibroblasts treated by different drugs.

	**Untreated**	**Latrunculin A**	**Blebbistatin**	**Calyculin A**	**CK666**
*f*_T_ (kHz)	49.17	13.08	14.06	160.7	84.98
*f*_crossover_ (kHz)	68.11	21.4	17.98	240.32	67.80

**Fig. 5.**
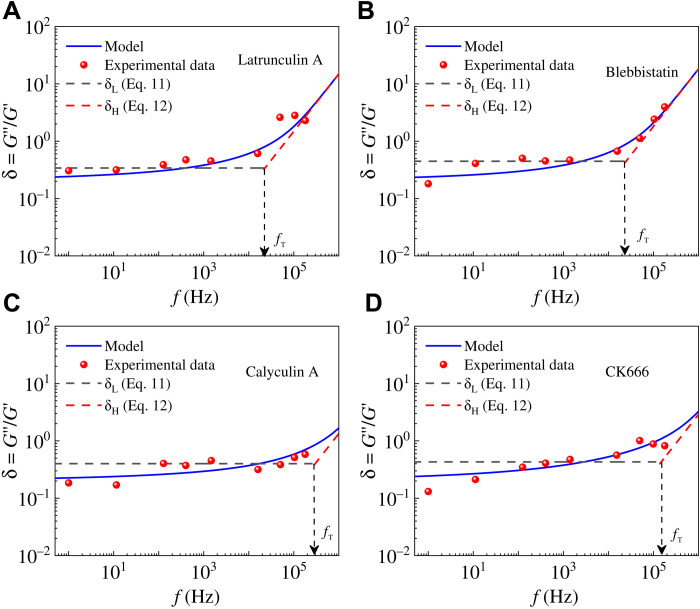
The transition frequency of cells treated with different drugs. (**A**) Latrunculin A, (**B**) blebbistatin, (**C**) calyculin A, and (**D**) CK666. Experimental data are obtained from (*10*).

From the results in Figs. 3B and 5, the transition frequency calculated by our self-similar hierarchical model measures the critical point beyond which the value of δ increases significantly with frequency. The trend of the transition frequency is similar to that of the crossover frequency defined by Staunton *et al*. (*29*), as shown in Table 2. Differently, our defined transition frequency is calculated by the mechanical properties (*E*_1_, *E*_2_, *E*_3_, and η) that can be obtained from experimental results in the low-frequency ranges. In contrast, experimental determination of the crossover frequency *f*_T_ can be quite costly. As summarized in Tables 1 and 2, the variations of cell’s mechanical properties, induced by different drugs, can be well characterized by the proposed model and defined transition frequency.

### Dynamical mechanical changes of benign MCF10A and malignant MCF7 cancer cells

**Fig. 6.**
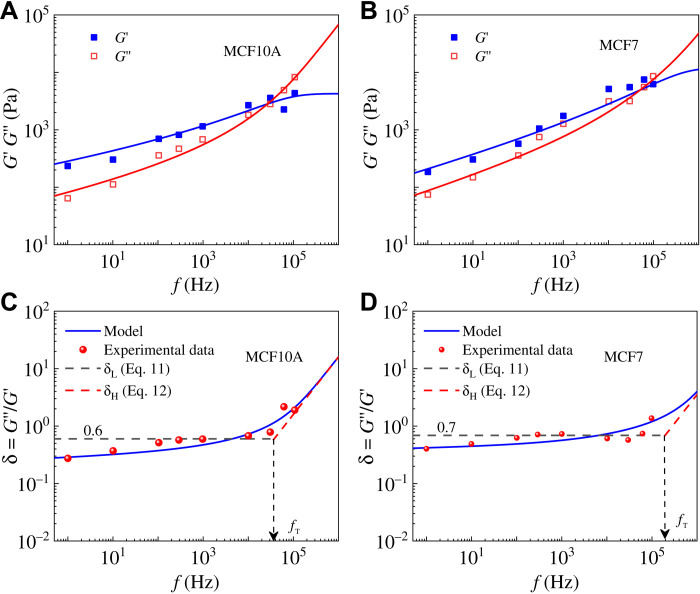
The self-similar hierarchical model agrees well with experimental results from (*10*). Frequency-dependent storage and loss moduli of (**A**) MCF10A and (**B**) MCF7 cancer cells. Frequency-dependent loss tangents δ of (**C**) benign MCF10A and (**D**) malignant MCF7 cancer cells.

Experiments have shown that benign and malignant cancer cells exhibit remarkably distinct rheological properties (*27*, *33*–*35*), especially at high frequencies (*10*). Here, we use the proposed model to explore mechanisms that underlie the differences in mechanical properties of benign MCF10A and malignant MCF7 cancer cells. As shown in Fig. 6, our model can fit well with the complex moduli and the loss tangents δ of benign MCF10A and malignant MCF7 cancer cells on a vast frequency scale. The obtained mechanical properties are listed in Table 3, and the differences can be understood as follows. The viscous coefficient η of malignant cancer cells is less than benign ones, which is consistent with previous reports (*34*, *36*). Because of the deterioration of malignant MCF7 cancer cells, their cytoskeleton tends to disorganize and depolymerize (*36*), and as a result, the elastic stiffness *E*_3_ sharply reduces from 2.46 to 0.01 kPa. For MCF7 cells, the connecting microfilaments are abnormal, and the stress fibers are detached from the cytoplasm and the cell membrane (*37*, *38*). Consequently, the cytoskeleton becomes highly disorganized, resulting in a sharply reduced value of *E*_3_. It is noted that *E*_3_ exhibits higher sensitivity than other parameters in the self-similar model (see Table 3). In addition, when Rigato *et al*. (*10*) used their double power-law model to describe the rheological behaviors of MCF7 and MCF10A cells, the difference between the elastic stiffnesses of the two types of cells at high frequencies is also nearly 20 times. On the other hand, the total stiffness *E*_sum_ = *E*_1_ + *E*_2_ + *E*_3_ of MCF7 cells is only about twice as large as that of MCF10A cells. The elastic stiffness *E*_1_ of MCF7 cells is far larger than that of MCF10A cells, because the depolymerized components contribute to the stiffness of the cytoplasm (see Table 3). The transition frequency of malignant MCF7 cells is far higher than that of MCF10A cancer cells (see Table 3), which is in line with the trend of crossover frequency (*29*) but with a much higher ratio ~5.2 than that of the crossover frequency ~1.9. This indicates that our defined transition frequency is capable of differentiating between cells in different states. It can be seen from Eq. 13 that the transition frequency is calculated by using mechanical properties obtained in the low-frequency range only. This is distinctly different from the crossover frequency that is obtained by the intersection of the storage and loss modulus at relatively high frequencies in experiments. The changes in mechanical properties of cells during carcinogenesis (*10*, *29*) show potential for application as new markers for cancer diagnosis, although there is still no consensus on the formation mechanism of these changes. Linking mechanical parameters (*E*_1_, *E*_2_, *E*_3_, η, *f*_T_, etc.) with biological factors can draw us closer to developing mechanics-based markers for early cancer diagnosis.

**Table 3.** Mechanical parameters of benign MCF10A and malignant MCF7 cancer cells obtained by our model.

	η **(Pa·s)**	*E*_1_ **(kPa)**	*E*_2_ **(kPa)**	*E*_3_ **(kPa)**	*f*_T_ **(kHz)**	*f*_crossover_ **(kHz)**
MCF10A	0.011 ± 10^−4^	1.74 ± 0.20	2.27 ± 0.43	2.46 ± 0.34	37.1	26.58
MCF7	0.007 ± 10^−4^	10.95 ± 0.56	2.00 ± 0.20	0.01 ± 0.05	192.5	51.40

### Comparison with other models

Emad *et al*. (*19*) used a poroelastic theory to describe the relaxation behavior of cells, yet the relationship between poroelastic materials and the power-law rheology is not clear. The creep response of poroelastic materials can be simplified as (see note S1)

$$u^{o}(t)=\frac{2t^{0.5}}{\sqrt{\pi }l}$$ (14)

where *l* is a constant related to the permeability coefficient and percentage of solids. A comparison of the numerical solutions (*39*) and our simplified equation (Eq. 14) is shown in fig. S3 (see note S1), where the creep response of poroelastic materials shows a power-law dependence on time with an exponent of 0.5. Because the power-law exponent of complex modulus over frequency is the same as that of creep compliance over time (see note S2), the power-law exponent of poroelastic materials in the frequency domain is also 0.5. The power-law exponent greater than 1.0 in the double power-law exponent model (*10*) may come from other factors rather than the effect of poroelasticity. When α = 0.5, one has δ = tan (απ/2) = 1, which can be considered a critical point of the cell, with a more solid-like behavior when δ < 1 and a more fluid-like when δ > 1 (*17*, *29*, *40*).

A four-element viscoelastic model (see fig. S5A in note S3) has been proposed to characterize the viscoelastic behavior of cells (*15*). Nicolas *et al*. (*14*) used it to depict the power-law creep properties of cells in a certain time range. Typical curves of the complex modulus of the four-element viscoelastic model are given in fig. S5B. It can be seen that the storage and loss moduli show a power-law dependence on frequency only in a certain frequency range. However, as ω approaches infinity, the storage modulus of this model approaches a constant, while the loss modulus approaches 0 (see note S3). As ω approaches 0, the complex modulus of this model is close to that of the dashpot (η_2_), with the storage modulus being 0 and the loss modulus being ωη_2_ (eq. S22). These rheological predictions are at odds with the behaviors of real cells, and this model cannot describe the cell’s rheological properties in a wide frequency range.

The SGR model can reproduce the weak power-law frequency dependence of storage and loss moduli in the low-frequency range with the same power-law exponent that relies on the mean-field noise temperature. However, the loss modulus of the SGR model tends to 0 in the high-frequency range (*18*), which is not consistent with the experimentally observed behavior of living cells.

As summarized in many experiments (*9*, *10*, *12*, *29*), the storage and loss moduli exhibit a weak power-law dependence on the frequency with similar power-law exponents within the low-frequency range. In the high-frequency range, the storage modulus tends to a constant (i.e., power-law rheology with an exponent of 0), while the loss modulus shows a power-law dependence on frequency with an exponent of 1.0. These rheological characteristics of cells cannot be captured by various models including the simple power-law model, the SGR model, the poroelastic model, the four-element model, etc. (see Table 4). In contrast, our self-similar hierarchical model, capturing the multilevel structural features of cells, can naturally reproduce the power-law rheological behavior of cells in different frequency scales, and the defined transition frequency succinctly describes the transition of cell’s rheological characteristics from lower to higher frequencies.

**Table 4.** Comparison of cell’s rheological characteristics predicted by different models.

**Model**	**Low frequency**		**High frequency**		**Power-law exponent** α
	***G*′**	***G*″**	***G*′**	***G*″**	
Poroelastic	*G*^′^ ∝ ω^0.5^	*G*^″^ ∝ ω^0.5^	*G*^′^ ∝ ω^0.5^	*G*^″^ ∝ ω^0.5^	0.5
Four-element	*G*^′^ ~ ω^α^	*G*^″^ ~ ωη	$\underset{\omega \to \infty }{\text{lim}}G\prime =\text{constant}$	$\underset{\omega \to \infty }{\text{lim}}G\prime \prime =0$	Approximate power-law (*14*)
SGR	*G*^′^ ∝ ω^α^	*G*^″^ ∝ ω^α^	$\underset{\omega \to \infty }{\text{lim}}G\prime =\text{constant}$	$\underset{\omega \to \infty }{\text{lim}}G\prime \prime =0$	Depending on the mean-field noise temperature (*18*)
Present self-similar hierarchical	*G*^′^ ∝ ω^α^	*G*^″^ ∝ ω^α^	$\underset{\omega \to \infty }{\text{lim}}G\prime =\text{constant}$	$\underset{\omega \to \infty }{\text{lim}}G\prime \prime =\omega \eta$	0.125 to 1.0
Living cells	*G*^′^ ∝ ω^α^	*G*^″^ ∝ ω^α^	$\underset{\omega \to \infty }{\text{lim}}G\prime =\text{constant}$	$\underset{\omega \to \infty }{\text{lim}}G\prime \prime =\omega \eta$	0.1 to 1.0

## DISCUSSION

Living cells exhibit distinctly different power-law rheological characteristics at different frequency scales. At low frequencies, both storage and loss moduli of cells show a weak power-law dependence on frequency with similar exponents. At high frequencies, the storage modulus of cells tends to a constant, while the loss modulus becomes linearly proportional to frequency. On the basis of the multilevel structural features of cells, we have proposed a self-similar hierarchical model that can naturally capture a cell’s rheological characteristics spanning several orders of magnitude of frequency. Our model shows that the cell’s rheological responses in a wide frequency range can be divided into three regions, depending on the value of the loss tangent. In the low-frequency range (region I), the complex moduli of most cells exhibit a weak power-law dependence on frequency, and the loss tangent δ is approximately a constant. In experiments (*29*), the storage modulus of cells exhibits a nearly flat plateau region at very low frequencies, corresponding to a relatively small power-law exponent. As the frequency increases (region II), the loss modulus *G*″ shows a greater power-law dependence on frequency than the storage modulus *G*′. When the frequency is sufficiently high, the loss tangent δ > 1 (region III), and the loss modulus shows a greater power-law dependence on frequency, while the storage modulus converges to a constant. Our self-similar hierarchical model realizes the rheological behavior of different types of cells in different frequency ranges (from 10^−2^ to 10^5^ Hz), which are in broad agreement with experiments (*9*, *10*, *12*, *25*). Furthermore, the drug-induced cytoskeletal differences can also be characterized by changes in the mechanical parameters in our model. A transition frequency, defined on the basis of the mechanical parameters in the model, can well describe the cell’s rheological differences under different frequency ranges for different cell states or types. The transition frequency of malignant cancer cells is nearly five times higher than that of benign cancer cells. It should be mentioned that our model is a theoretical model, while experimental conditions are more complicated and various other factors (e.g., solvent viscosity) could influence the results. Nevertheless, our model captures dynamical mechanical characteristics of cells under different frequency scales and may provide a theoretical foundation to characterize cells’ biological changes (e.g., drug treated and canceration) through their rheological properties.

## MATERIALS AND METHODS

### Data analysis and fitting

The complex moduli of cells were fitted by using Eq. 4. As the complex modulus is a complicated nonlinear function, we first estimate the parameters of the fitting function as initial values and use iterative optimization to find the locally optimal parameters, which are derived by using the nlinfit function in MATLAB. After finding the local optimal parameters in one iteration, we then use these values as the initial values to find their values for the next iteration, until the difference between two iterations is less than 1%. All data processing and analysis were performed in MATLAB 2016b (MathWorks).

### Numerical solution of the complex modulus

The theoretical solution for the complex moduli gives the storage and loss moduli according to Eq. 4. The numerical solution is cumulative according to the rules for the series and parallel connection of mechanical elements, as shown in Fig. 1. The number of elements in this work is taken as 5000. The recurrence relations of the 1st-level hierarchy in numerical analysis are

$$\begin{array}{c} G_{1}^{*}(1)=E_{1}\\ G_{1}^{*}(2)=E_{1}+i\omega \eta \\ \vdots \\ G_{1}^{*}(2n-1)=1/(1/G_{1}^{*}(2n-2)+1/E_{1})\\ G_{1}^{*}(2n)=G_{1}^{*}(2n-1)+i\omega \eta \end{array}$$

The recurrence relations of the 2nd- and 3rd-level hierarchies can be obtained similar to those of the 1st-level hierarchy. All data processing and analysis were performed in MATLAB 2016b (MathWorks).
