# Supplementary material for: Frequency-dependent transition in power-law rheological behavior of living cells
Source: Sci Adv. 2022 May 6;8(18):eabn6093. doi: 10.1126/sciadv.abn6093 (PMC9075802; doi:10.1126/sciadv.abn6093)
Supplement: Supplementary file 1 — Supplementary Text Figs. S1 to S5 [file sciadv.abn6093_sm.pdf]

Science Advances
AAAS

Supplementary Materials for

Frequency-dependent transition in power-law rheological behavior of living cells

Jiu-Tao Hang, Guang-Kui Xu*, Huajian Gao*

*Corresponding author. Email: guangkuixu@mail.xjtu.edu.cn (G.-K.X.); huajian.gao@ntu.edu.sg (H.G.)

Published 6 May 2022, *Sci. Adv.* **8**, eabn6093 (2022)
DOI: 10.1126/sciadv.abn6093

This PDF file includes:

Supplementary Text
Figs. S1 to S5

Supplementary Text

Supplementary Note 1. The power-law rheology of poroelastic materials

Mow et al. *(39)* obtained the creep response of poroelastic materials by solving the corresponding governing equations for their displacement response under a step stress. Here we used their results for further simplification to explore the power-law rheological response of poroelastic materials. The normalized displacement response of poroelastic materials under a step force is *(39)*

$$u^0(t)=\frac{2f_0}{l^2}\sum_{n=0}^{\infty}\frac{1}{P_n^2}\left(1-e^{-P_n^2 t}\right), \tag{S1}$$

where $l^2=(1+2\alpha_0)/H$, $P_n=(2n+1)\pi/2l$, α_0 is the initial percentage of solid content, H is a constant related to the permeability coefficient and percentage of solids *(19)*, and f_0 is a constant related to the step force *(39)*. Here, we re-write the formula $\sum_{n=0}^{\infty}\frac{1}{P_n^2}\left(1-e^{-P_n^2 t}\right)$ into the form of $\sum_{i=0}^{\infty}\tau_i\left(1-e^{-t/\tau_i}\right)$ where $\tau_i=4l^2/\left(2(N-i)+1\right)^2\pi^2$. This expression is consistent with that of the creep compliance of the generalized Kelvin-Voigt viscoelastic model (Fig. S1).

By taking l with different values, we analyze the distribution of $\log(\tau)$, as shown in Fig. S2. It is found that the occurrence frequency of τ decreases linearly with relaxation time in the log-log coordinate system with a slope of –0.5. We can examine the distribution of τ under different values of l by conducting statistics on $\log(\tau)$, and obtain

$$\frac{df_\tau}{d\tau}=\frac{df_\tau}{d(\log\tau)}\frac{d(\log\tau)}{d\tau}=\frac{df_\tau}{d(\log(\tau))}\tau^{-1}\frac{1}{\ln 10}\propto\tau^{-\frac{3}{2}}. \tag{S2}$$

Next, we explore the origin of the power-law distribution of τ by using the probability analysis method. Supposing *x* is uniformly distributed, the distribution function is $P(X=x)=c$, where c is a constant, when $x>0$. The random variable Y is a measurable and continuous function of the random variable X, $Y=a/(2X+1)^2$. The probability distribution function of Y is:

$$F_Y(y)=P\left\{\frac{a}{(2X+b)^2}\le y\right\}=\int_{(\sqrt{a/y}-b)/2}^{X_{\max}}f_X(x)\,dx=1-f_X(x)\left(\sqrt{a/y}-b\right)/2. \tag{S3}$$

From the above, one can obtain the probability density function of Y as:

$$f_Y(y)=F_Y'(y)=0.25\sqrt{a}f_X(x)y^{-\frac{3}{2}}. \tag{S4}$$

By taking N as the maximum value of n and $f_X(X=n/N)$ with $X\sim U(0,1)$, the relaxation time is written as

$$\tau = \frac{a}{(2n+1)^2} = \frac{a}{N^2}\frac{1}{(2n/N+1/N)^2} = \frac{a}{N^2}\frac{1}{(2\mathrm{X}+1/N)^2}, \tag{S5}$$

where $a = 4l^2/\pi^2$. By substituting Eq. (S5) into Eq. (S4), we obtain the probability density function of relaxation time τ as:

$$P(\tau) = \frac{l\tau^{-\frac{3}{2}}}{2N\pi}. \tag{S6}$$

The above results obtained from mathematical statistics (Eq. (S6)) are consistent with those from numerical solutions (Fig. S3), which verifies that $P(\tau)$ is proportional to $\tau^{-3/2}$. Then, we can write the sum as an integral of

$$\frac{1}{N}\frac{2}{l^2}\sum_{n=0}^{\infty}\tau\left(1-e^{-t/\tau}\right) = \frac{2}{l^2}\int_0^{\infty}\tau P(\tau)\left(1-e^{-t/\tau}\right)d\tau. \tag{S7}$$

$$\begin{aligned}\frac{2}{l^2}\sum_{n=0}^{\infty}\tau\left(1-e^{-t/\tau}\right) &= \frac{2}{l^2}\int_0^{\infty}\tau\frac{l\tau^{-1.5}}{\pi}\left(1-e^{-t/\tau}\right)d\tau \\ &= \frac{1}{l\pi}\int_0^{\infty}\tau^{-0.5}\left(1-e^{-t/\tau}\right)d\tau \\ &= \frac{1}{l\pi}\left(2\left(1-e^{-t/\tau}\right)\sqrt{\tau} - 2\sqrt{\pi t}\,Erf\left[\sqrt{t/\tau}\right]\Big|_{\tau=0}^{\tau=\infty}\right) \\ &= 2t^{0.5}/l\sqrt{\pi}\end{aligned}. \tag{S8}$$

It should be noted that the upper limit of $u^o(t)$ in Eq.(S1) is 1.0.

Through the above derivation of probability statistics (Eqs.(S1–S8)), we get the simplified expression of the creep response of poroelastic materials:

$$u^o(t) = \frac{2f_0t^{0.5}}{\sqrt{\pi}l}. \tag{S9}$$

Comparison of the simplified expression (Eq. (S9)) with the original expression (Eq. (S1)) in Fig. S3 shows that the power-law exponent of the creep compliance on time is 0.5. Since the power-law exponent of the creep compliance over time coincides with that of the complex modulus over frequency (see details in Supplementary Note 2), the power-law exponent of the complex modulus of the poroelastic model is also 0.5. Therefore, the power-law exponent greater than 1.0 in the double power-law exponent model *(10)* may come from other factors rather than the effect of poroelasticity.

We also established a cellular finite element model composed of poroelastic materials with different conformations (see Fig. S4 (a, b)) to further examine Eq. (S9). If the cell has a variable cross-section structure, the power-law exponents of poroelastic materials are all 0.5 for different pore pressures (Fig. S4 (a)). If the cell takes a cylindrical structure, we also get a power-law exponent of 0.5 for different pore pressures (Fig. S4 (b)). In summary, the creep response of the finite element model composed of poroelastic materials follows a power-law dependence on time with an exponent of 0.5, which further validates our simplified expression Eq.(S9).

Supplementary Note 2. The relationship between complex modulus and creep compliance of power-law rheology

Here, we derive the relationship between the power-law exponent of the complex modulus in the frequency domain and that of the creep compliance in the time domain. When a given material is subjected to a varying stress $\sigma(t)$, the induced strain $\varepsilon(t)$ is related to $\sigma(t)$ in the linear regime by

$$\varepsilon(t) = J(t)\sigma(0) + \int_0^t J(t-\tau)\frac{d\sigma(\tau)}{d\tau}d\tau\,, \tag{S10}$$

When a given material is subjected to a varying strain $\varepsilon(t)$, the induced stress $\sigma(t)$ is related to $\varepsilon(t)$ in the linear regime by

$$\sigma(t) = G(t)\varepsilon(0) + \int_0^t G(t-\tau)\frac{d\varepsilon(\tau)}{d\tau}d\tau\,, \tag{S11}$$

where $J(t)$ is the creep compliance and $G(t)$ is the relaxation modulus.

Laplace transform of Eq. (S10) and Eq. (S11) yields

$$\tilde{\varepsilon}(s) = s\tilde{J}(s)\tilde{\sigma}(s)\,, \tag{S12}$$

$$\tilde{\sigma}(s) = s\tilde{G}(s)\tilde{\varepsilon}(s)\,. \tag{S13}$$

By combining Eq.(S12) and Eq.(S13), the relationship between creep compliance and relaxation modulus can be obtained as

$$\tilde{G}(s)\tilde{J}(s) = \frac{1}{s^2}\,. \tag{S14}$$

Considering Eq.(S14) and the definition of complex modulus $G^*(s) = \tilde{\sigma}(s)/\tilde{\varepsilon}(s)$, the relationship between complex modulus and creep compliance is:

$$G^*(s)\tilde{J}(s) = \frac{1}{s}\,. \tag{S15}$$

If the creep compliance exhibits a power-law dependence on time, one has:

$$J(t) = At^{\alpha}\,, \tag{S16}$$

where A is a prefactor. The Laplace transform of the creep compliance is:

$$\tilde{J}(s) = \frac{A\Gamma(\alpha+1)}{s^{\alpha+1}}\,. \tag{S17}$$

Then, the complex modulus in Laplace space can be obtained:

$$G^*(s) = \frac{1}{s\tilde{J}(s)} = \frac{s^{\alpha}}{A\Gamma(\alpha+1)}\,. \tag{S18}$$

The storage and loss moduli over the angular frequency are expressed as:

$$G'(\omega) = \frac{\omega^{\alpha}\cos(\alpha\pi/2)}{A\Gamma(\alpha+1)}\,, \tag{S19}$$

$$G''(\omega) = \frac{\omega^\alpha \sin(\alpha\pi/2)}{A\Gamma(\alpha+1)}. \quad \text{(S20)}$$

Supplementary Note 3. Rheological response of a 4-element viscoelastic model

As illustrated in Fig. S5 (a), a 4-element model can be described by a 3-element model (G_R) and a dashpot with viscous coefficient η_2 *(14, 15)*. The complex modulus of the 3-element model is expressed by $G_R^* = (\mathrm{i}\omega\eta_1 G_{R1})/(G_{R1} + \mathrm{i}\omega\eta_1) + G_{R2}$, where G_{R1} and G_{R2} represent the elastic stiffnesses of the two springs, and η_1 represents the viscous coefficient of the dashpot in the 3-element model. As ω goes to infinity, the complex modulus of the 4-element model is:

$$\lim_{\omega\to\infty} G^* = \frac{G_R^*(\mathrm{i}\omega\eta_2)}{G_R^* + \mathrm{i}\omega\eta_2} = \frac{G_R^*}{1 + G_R^*/\mathrm{i}\omega\eta_2} = G_R = G_{R1} + G_{R2}. \quad \text{(S21)}$$

As ω goes to be 0, the complex modulus of the 4-element model is:

$$\lim_{\omega\to 0} G^* = \frac{G_R^*(\mathrm{i}\omega\eta_2)}{G_R^* + \mathrm{i}\omega\eta_2} = \mathrm{i}\omega\eta_2. \quad \text{(S22)}$$

Supplementary Note 4. The physical meanings of each parameter in the self-similar hierarchical model

Based on the structural characteristics of the cells, we propose a self-similar hierarchical model to capture their rheological behavior. Since the cytoplasm is ubiquitous, its spatial component can be discretized and regarded as springs immersed in a viscous fluid, as shown in Fig. 1. The cytoplasm is treated as the 1st level hierarchy, which fills the entire cell. A single cytoskeletal filament can be discretized into a series of springs, with each node connected to the cytoplasm (the 1st level hierarchy). Then, each cytoskeletal filament embedded into the cytoplasm is organized as the 2nd level hierarchy with the 1st level hierarchy as a building block, as shown in Fig. 1. Because there are many cytoskeletal filaments with different lengths and orientations in the cell, the whole structure network is treated as a large number of cytoskeletal filaments (the 2nd level hierarchy) connected by springs. In this way, the entire cell is considered the 3rd level hierarchy with 2nd level as a building block (see Fig. 1). In this way, the previous level hierarchy is embedded into the current level as a building block, making each level similar in form and the whole system self-similar.

In our model, there are a total of four parameters representing the mechanical properties of cells: E_1 represents the effective stiffness of the cytoplasm, η the effective viscosity of the cytoplasm, E_2 the effective stiffness of the cytoskeletal filaments, and E_3 the effective stiffness of the transverse expansion of the cytoskeletal network. Among these, E_1 and η can be obtained through experimental measurements on cytoplasm while the sum of E_1, E_2 and E_3 representing the total elastic stiffness of cells can be measured by, e.g. indentation experiments. The total elastic moduli obtained from indentation experiments are 5.0 ~ 11.2 kPa for 3T3 Fibroblasts cells *(30, 31)* and 0.2 ~ 2 kPa for bronchial epithelial cells *(32)*, which are in good agreement with our results (11.23 kPa for 3T3 Fibroblasts cells and 1.72kPa for bronchial epithelial cells).

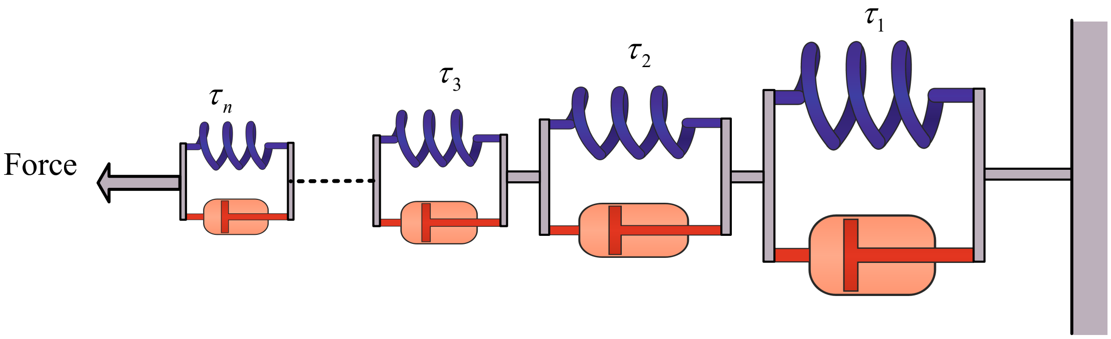

Fig. S1. The generalized Kelvin-Voigt viscoelastic model. Schematic representation of a generalized Kelvin-Voigt viscoelastic model with rheological properties determined by the distribution of relaxation time (τ_i).

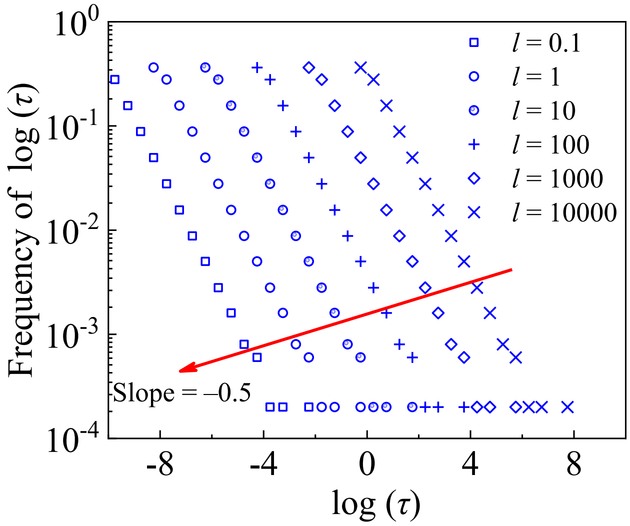

Fig. S2. Distribution of log (τ). Frequency of $\log(\tau)$ under different values of l when $N = 5000$.

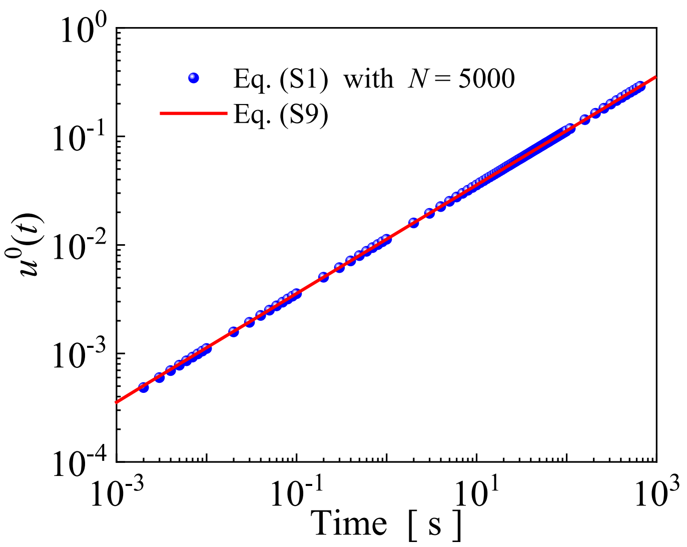

Fig. S3. Creep response of poroelastic materials shows a power-law dependence on time with an exponent of 0.5. Comparison of numerical and simplified solutions for the creep response of poroelastic materials by taking $l = 1000$. The scatter data and solid line represent the numerical results (Eq. (S1)) and the simplified expression (Eq. (S9)), respectively.

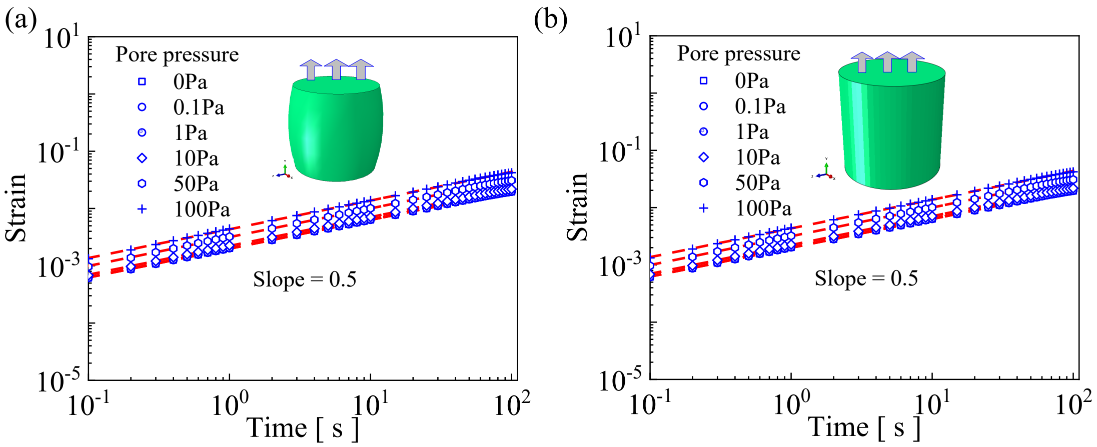

Fig. S4. Creep responses of the finite element model of cells show a power-law dependence on time with an exponent of 0.5. (a) Creep responses of the cellular finite element model with a variable cross-section under different pore pressures exhibit the form of $\sim t^{0.5}$. (b) Creep responses of the cellular finite element model with a fixed cross-section under different pore pressures exhibit the form of $\sim t^{0.5}$.

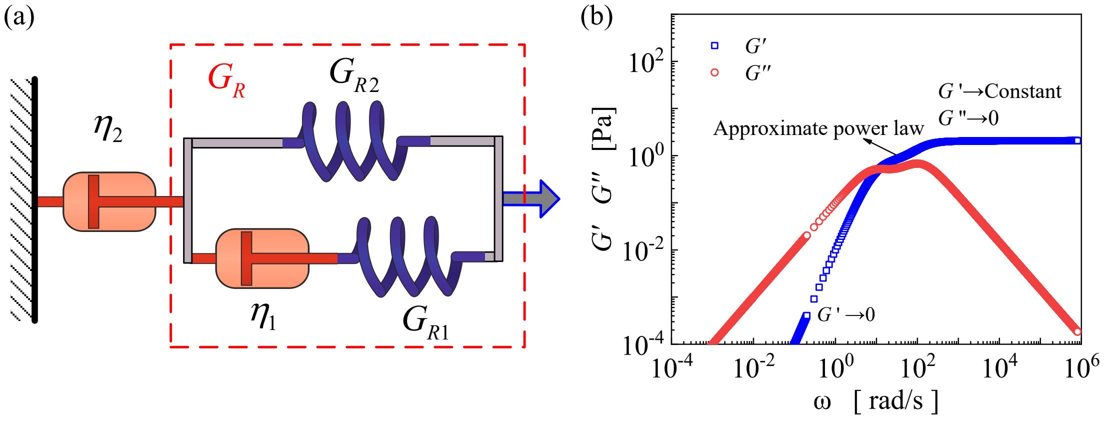

Fig. S5. Rheological responses of a 4-element viscoelastic model. (a) Schematic diagram of the 4-element viscoelastic model. G_{R1} and G_{R2} represent the elastic stiffnesses of the two springs, and η_1 and η_2 the viscous coefficients of the two dashpots. (b) Typical curves of the complex modulus of the 4-element viscoelastic model with respect to the angular frequency.
